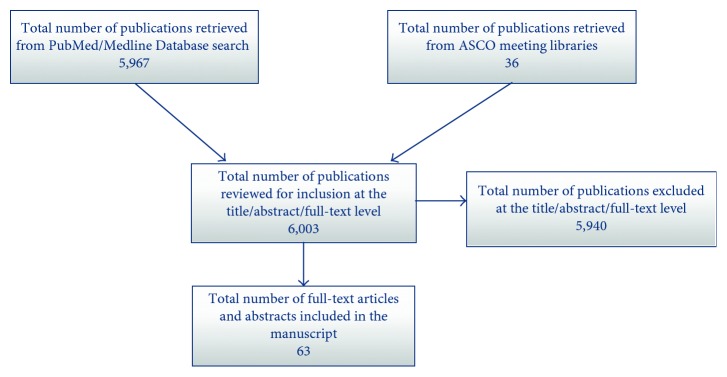# Immune-Related Concepts in Biology and Treatment of Germ-Cell Tumors

**DOI:** 10.1155/2018/3718165

**Published:** 2018-03-13

**Authors:** Michal Chovanec, Ugo De Giorgi, Michal Mego

**Affiliations:** ^1^2nd Department of Oncology, Faculty of Medicine, Comenius University, National Cancer Institute, Bratislava, Slovakia; ^2^Divison of Hematologya and Oncology, Indiana University Melvin and Bren Simon Cancer Center, Indianapolis, IN, USA; ^3^Medical Oncology Department, Istituto Scientifico Romagnolo per lo Studio e la Cura dei Tumori (IRST), IRCCS, Via Piero Maroncelli 40, 47014 Meldola, Italy

## Abstract

Germ-cell tumors (GCTs) are highly curable with chemotherapy. Salvage chemotherapy or surgery can cure a proportion of patients, but the ones failing these treatments will die of their disease in the young age. Immune checkpoint pathways are emerging as powerful targetable biomarkers, and a significant preclinical and clinical research is underway to widen our knowledge and expand the treatment possibilities with immune therapy. The concept of immune modulation that was currently adopted in many solid tumors is understudied in GCTs. Herein, we summarize the current knowledge of published literature discussing the immune mechanisms and immune therapy in GCTs.

## 1. Introduction

The success story of curing germ-cell tumors (GCTs) established a cisplatin-based chemotherapy as a mainstay for achieving >95% relative overall survival rate [1]. Unfortunately, chemotherapy is not a universal cure for all patients with metastatic disease. About 40–80% of patients with relapse after initial chemotherapy will fail the salvage treatment, and their prognosis is dismal [2–4]. Researchers have spent limitless effort to further advance the anticancer treatment with the most recent substantial achievement being the discovery of modern immune therapy in numerous solid cancers. GCTs are traditionally referred to as “a model for cure” with chemotherapy; however, a scientific inquiry has risen, whether the immune mechanisms may be as important in GCTs as it is in melanoma, lung cancer, kidney cancer, or others. The failure of chemotherapy in substantial proportion of GCT patients creates a therapeutic dilemma for many decades with no major advancement since the introduction of the salvage chemotherapy. Therefore, the question whether immunity plays a significant role in the pathogenesis of GCTs is critical to answer, allowing to derive implications for future development in the treatment of this unique solid malignancy. Herein, we summarize the current knowledge of the literature to provide the insights into the immune biology of GCTs.

## 2. Literature Search

We performed a literature search of the PubMed/Medline database and meeting libraries of American Society of Clinical Oncology (ASCO) and ASCO Genitourinary Cancers Symposium for publications with the terms “testicular germ cell tumors,” “immunity,” “immune,” “immunotherapy,” “tumor infiltrating lymphocytes,” “TIL,” “inflammation,” “cytokines,” “check-point.” Combinations of these key words were used for comprehensive search as outlined in Figure 1. The search of literature was performed on September 1, 2017. Original full-text articles published in English were reviewed, and the reference lists of key articles were further evaluated. We did not limit our search by the years of publication. Our search was conducted according to the Preferred Reporting Items for Systematic Review and Meta-Analysis (PRISMA) statement. Identified reports were reviewed according to the Consolidated Standards of Reporting Trials (CONSORT) criteria. The search resulted in overall 6003 publications. Sixty-three publications were selected for inclusion in our review article. The outline of the literature search is summarized in Figure 1.

## 3. Immune-Cell Infiltration, Cytokine Signaling, and Genomic Underpinnings

The observation of immune reaction induced by GCTs evidenced by lymphocytic and granulomatous intratumoral infiltrates dates as early as 1964 and was published by Marshal and Dayan from Bernhard Baron Institute of Pathology in London [5]. Rich infiltration with immune cells in testicular GCTs, particularly in seminomas, was observed by number of other earlier studies [6–11], suggesting an involvement of the immune system in GCT biology. The immune-cell characterization of seminoma by Bols et al. has shown tumor infiltrating lymphocytes (TILs) that included CD3+ and T-memory cell populations, while B-cells and plasma cells were present less frequently. Seminoma in situ (germ-cell neoplasia in situ adjacent to seminoma) was infiltrated mainly by CD4+ and CD8+ TILs and then followed by B-cells, dendritic cells, NKcells, and macrophages. This study was also among the first ones to provide data about prognostic significance of TILs, where authors showed that lower TIL count was associated with the risk of relapse [9]. Hvarness et al. provided similar observation of immune microenvironment in germ-cell neoplasia in situ describing macrophages, CD8+T-cells, CD450R0+ T-cells, and B-cells present in the GCNIS tissue [12]. T-lymphocytes and macrophages were also documented in an embryonal carcinoma [8]. Further observations presented data of characteristic cytokine signaling encompassing tumor tissue with immune-cell infiltrates. Klein and colleagues observed a presence of B-cells and dendritic cells in GCNIS, accompanied by high level of transcripts of several proinflammatory (IL6, IL-1*β*, and TNF-*α*), anti-inflammatory (TGF-*β*1), Th1-driven (IL-2 and IFN-*γ*) cytokines, and chemokines (CXCL-13, CXCL-10, and CCL-5) [13]. In vitro experiments were conducted on seminoma-derived T-cam2 and peripheral blood mononuclear cell (PBMC) coculture. PBMC exhibited a robust increase in the production of IL1*β*, TNF-*α*, TGF-*β*, and CCL5 transcript levels after direct, but not after indirect, contact with T-cam2 cells. PBMC previously stimulated with the mitogen phytohemaglutinin also exhibited significantly higher expression of IL-1*β*, IL-6, and IFN-*γ*. Moreover, authors provided surprising observation of the natural production of IL-6 by T-cam2 cell lines [14]. A specific cytokine signature was also observed as a response to murine testicular teratoma. Two cytokines, IL-6 and IL-10, were the most abundant allowing for subsequent promotion of humoral immune response [15]. Cytokine signatures observed to date position IL-6 signalling as one of the proposed central proinflammatory mechanisms in GCTs. Furthermore, a study by Purdue et al. that evaluated single nucleotide polymorphisms (SNPs) in immune function genes uncovered that SNPs in *TGFB*1, *LTA/TNF*, and others (*IL*2, *IFNGR*2, and *IL*10) may be responsible for an increase in the susceptibility for GCTs [16]. Recent study described distinct molecular signatures of immune cells specifically for seminoma showing elevation of expression signatures for B-cells, cytotoxic T-cells, Th17 cells, and T-regulatory cells (T-reg). This increase was associated with an increase in specific cytokines and immune checkpoints (CTLA4, LAG3, and PD-L1). Interestingly, there was no correlation between immune cells and load of mutations, neoantigens, or detected viruses, which confirms observations obtained from TCGA (The Cancer Genome Atlas) datasets [17–19]. The strongest association of growth in immune-cell populations was observed with activating mutations in *KIT* together with increased expression profiles of *KIT* and *MHC class I* and *II* genes [17]. Another in vitro study tried to address IFN-*γ* produced by GCT cells (NTERA and NCCIT cell lines) as a potential treatment target by a specific blocking antibody but found that autocrine production of IFN-*γ* was insufficient to utilize the signaling blockade in favor of the cell inhibition [20].

However, the question of the specific role of immune surveillance in GCT development remains open. Authors from Denmark assessed the immune-cell infiltrate phenotype in GCNIS versus normal tissue and infertile testicular tissue and did not find differences in immune infiltrating cells in this region. Authors, thus, speculated that immune surveillance is not the critical factor for germ-cell development [12]. According to another study evaluating canine seminomas, T-reg TILs did not prove the critical role in the immune response in canine seminoma [21]. Androgen receptor mutation specific to Sertoli cells was discovered to result in loss of testicular immune privilege, a physiological barrier to prevent an immune response against germ-cell antigens. The experiment was performed on Sertoli cell-specific androgen receptor mutant mice, who exhibited higher intratesticular levels of IgG as compared with nonmutant mice, subsequently resulting into infertility [22]. It is not clear how this observation may fit into pathogenesis of GCTs. However, assessing the androgen-receptor associated signaling in patients with GCTs may shed more light into immune surveillance and its errors in patients with metastatic disease. Interesting observations were done in several studies showing that the incidence of GCTs significantly increases in the population of acquired immunodeficiency syndrome (AIDS) patients [23–25]. The number of events from these studies prevents from the conclusion whether acquired immunodeficiency alters GCT-specific survival in this patient population. Nevertheless, the raise in incidence of GCTs may provide indirect indications of the role of overall immune health in this disease development.

## 4. Immune-Related Biomarkers in GCTs

Despite the fact that we are not able to clearly assess the role of the immune surveillance in the development of GCTs, the prognostic role of TILs is clear. Data regarding new biomarkers, such as immune checkpoints, started to emerge recently as a result of astonishing clinical achievements of immune checkpoint inhibition in various malignancies.

Kersemaekers et al. suggested that FAS/FASL apoptotic signaling in GCT TILs may be a contributing factor to GCT development [26]. Another similar study by Schmelz, however, failed to replicate these results [27]. One of the new promising targets in different types of tumors is programmed-death-1 receptor (PD-1; CD279) and its ligand (PD-L1; B7-H1; CD274), which deliver inhibitory signals that regulate the balance between T-cell activation, tolerance, and immune-mediated tissue damage [28]. PD-1 is a member of the immunoglobulin superfamily and is expressed on double-negative T cells in thymus and on activated CD4+ T-cells, CD8+ T-cells, natural killer cells, B-cells, and monocytes [29]. It is primarily involved in modulating T-cell activity in peripheral tissues through interaction with its ligands PD-L1 and PD-L2 [9]. PD-L1 is expressed in different organs, including placenta, heart, lung, and liver as well as on activated T-cells, B-cells, dendritic cells, macrophages, and mesenchymal stem cells [30].

PD-L1 is expressed also on various tumor cells [29]. Expression of PD-L1 is an important process by which tumor cells suppress antitumor immunity in the tumor microenvironment [31]. Prognostic significance of PD-L1 expression on tumor cells was described in various malignancies [32–37], and the inhibition of PD-1/PD-L1 interaction has become an important landmark in cancer treatment. PD-1 and PD-L1 blocking antibodies have demonstrated clinical activity in several types of cancer including melanoma, nonsmall-cell lung cancer, renal cell cancer, ovarian cancer, and head and neck cancers [38].

The discovery of the PD-1 receptor and its ligand inspired Fankhauser et al. to conduct the first study in GCTs, which described the abundant expression of PD-L1 in seminoma and nonseminoma [39]. Our study explored the prognostic significance of PD-1/PD-L1 pathway in 140 patients with GCTs. We observed abundant expression of PD-L1 but not PD-1 on tumor cells and TILs (expressed in reverse manner on tumor and TILs) that correlated with poor risk clinical characteristics and survival. The low versus high expression of PD-L1 in tumor was associated with better survival (HR = 0.43; 95% CI 0.15–1.23; *P*=0.04 for OS) [40]. In contrast, high PD-L1 on TILs correlated with better survival (HR = 0.08; 95% CI 0.04–0.16; *P*=0.001 for OS). Based on these results, we developed a prognostic tool using PD-L1 on tumors and TILs independently of International Germ Cell Cancer Collaborative Group (IGCCCG) criteria [41]. Siska et al. performed a deep exploration of immune infiltrates in GCTs by immunohistochemistry and gene expression profiling and identified that activated T-cell infiltration correlated with seminoma and good prognosis. Advanced GCTs were associated with decreased T-cell and NK-cell signatures, while T-regs, mastocytes, and macrophages proved to be activated in patients with advanced stage disease. Authors also observed increased PD-L1 signalling in seminomas compared with nonseminomas using immunohistochemistry [42]. A study conducted on 102 patients from Japan evaluated the prognostic role of tumor-infiltrating neutrophils (TIN) by assessing CD66b+ TIN and found that they correlated with nodal and distant metastases, S stage, and nonseminoma histology. CD66b+ TIN were prognostic for progression-free and overall survival (*P* < 0.05 for both) [43]. The presence of inflammation in the cancer microenvironment may be identified with a simple clinical tool, the systemic immune inflammation index (SII), calculated as platelets × neutrophils/lymphocytes from the peripheral blood smear. SII proved to predict prognosis in several malignancies [18–22]. Chovanec et al. assessed the prognostic significance of SII in 240 GCT patients and discovered statistically significant differences in PFS and OS. Patients with lower levels of systemic inflammation, represented by lower SII, had significantly better prognosis compared with patients with high SII [44]. Preclinical research initiative by Terayama et al. aimed to evaluate testicular germ-cell specific autoimmunogenic antigens (AIs) in experimental mice with autoimmune orchitis. Authors immunized the serum of these mice with germ cells (nonmalignant), observing 11 specific AIs as a result. The results led to a speculation that these testis-specific AIs may provide a substrate for future research in targeting GCT novel targets [45]. The discovery of toll-like receptors (TLRs) has contributed to the expanding knowledge about the innate immune system. TLRs were proposed to contribute to cancer development [46]. TLRs 2, 3, 4, and 9 are expressed in testicular and GCT tissues; however, the expression in cancer tissue is significantly stronger [7]. Lin et al. published findings from a preclinical therapeutic trial in a mouse model using a conjugate of toll-like receptor 7 (TLR7) and OCT4. Mice were immunized with this novel agent and were subsequently challenged with the mouse embryonal carcinoma. Treatment with the conjugate resulted in significant release of IL12 and IFN-*γ* in vitro, and the significant increase in CD3+/CD8+ T cells occurred in vivo. Moreover, the rate of cytotoxicity in immunized mice was significantly higher, while the tumor growth decreased by 90% compared to the controls treated with OCT4 or TLR7 alone [47]. While experiments with animal models including animal cancer are difficult to interpret in the context of human biology, this study provides a pilot data of GCT immunogenicity. Vaccination with inhibin alpha was assessed and was effective against stromal cell tumors in preclinical in vitro and autochtonous mouse models but did not exhibit efficacy in GCTs [48]. Schreck et al. published an interesting observation of the loss of activation-induced cytidine deaminase (AID) in GCNIS and GCTs compared to the normal spermatocytes [49]. AID is normally involved in class-switch recombination of immunoglobulin genes in antigen-dependent B-cell maturation, suggesting that immune escape may be a mechanism participating in GCT development. This interpretation, however, may be biased and more studies are needed for validation.

## 5. Patterns in Cytokine and Immune-Cell Response to GCT and Its Treatment

Our research initiative investigated the patterns of cytokine signalling in peripheral blood in 79 patients with GCTs unveiling specific patterns in seminoma versus nonseminoma, nonpulmonary visceral metastases, and cerebral metastases and administered chemotherapy corresponded with the decline in the proinflammatory signature [50, 51]. These patterns generally suggest the tumor proangiogenic activity, the inhibition of immune response by low T- and NK-cell stimulation, the inhibition of T- and B-cell maturation, and the inhibition of chemoattraction and phagocytosis. The simultaneous immune stimulation and immune inhibition observed in our study was also described previously [51–53]. Further research has shown that increased prechemotherapy levels of cytokines promoting angiogenesis, tumorigenesis, immune stimulation, and chemoattraction correlated with shorter PFS and OS in 92 GCT patients [54]. Investigators from the University of Birmingham also observed a spontaneous CD4+ and CD8+ T-cell responses against cancer testis antigen in peripheral blood of patients with GCTs. The frequency of these T-cells substantially declined (by 89%) following orchiectomy for stage I GCTs [55].

## 6. Immune Therapy Efficacy and Clinical Trials

Early immunotherapy trials date to 1970s and 1980s. Undeniable efficacy of high-dose (HD) IL2 and HD IFN-*α* in selected patient population led to establishment of these treatments in malignant melanoma and kidney cancer. Anecdotal evidence existing in GCT patients is included in these clinical trials. The review summarized by Rosenberg et al. mentions 1 patient treated with HD IL2 + IFN- *α* and 1 patient treated with HD IL2 + lymphokine activated killer cells. No response was observed in either of them [56]. The concept of immune therapy has been abandoned or has never truly arisen because the excellent sensitivity of cisplatin-based chemotherapy provided substantially more powerful treatment results compared to other solid malignancies. Recent development of new biomarkers and novel immune therapies, however, raised questions, whether refractory GCTs may be candidates for immune checkpoint inhibition. A single-arm phase II study by Adra et al. from Indiana University recruited 12 patients with refractory GCTs to be treated with anti-PD1 inhibitor pembrolizumab. Although transient declines in tumor markers were noted in few cases, none of the patients had a radiographic response or a stable disease with this treatment [57]. Contradictory data to this trial exist from several case reports with platinum refractory GCTs treated with anti-PD1 agents (pembrolizumab or nivolumab) after salvage HD chemotherapy. These case reports describe possible responses in 3 out of 7 patients [58–60]. However, the interpretation of anti-PD1 treatment efficacy in these cases may be biased, as one of the patients received concomitant etoposide and the other two had only a short-term response to nivolumab. A single case report by Chi et al. provided evidence of ongoing partial remission with marker stabilization after treatment with nivolumab [61]. Based on the data from biomarker studies mentioned earlier in the article, we hypothesize that anti-PD-L1 treatment is more promising for patients with refractory GCTs and phase II trial with anti-PD-L1 inhibitor avelumab is being currently opened in Slovakia (NCT number not assigned yet). Another mechanism may be involved in immune regulation of GCTs, as proposed by Albany et al., who assessed guadecitabine, a demethylation agent, combined with cisplatin to reestablish a platinum sensitivity. A genome-wide analysis from their preclinical findings has shown that response to treatment was accompanied by activation of p53 together with the presence of immune-related pathways [62]. A phase I/II clinical study evaluating guadecitabine plus cisplatin in refractory GCTs is currently underway (NCT02429466).

## 7. Conclusion

GCTs and the immunity remains an understudied area. Existing evidence suggests active participation of immune system in the response to the presence of GCT and a certain level of evidence suggests its involvement in GCT development as well. Powerful biomarkers, such as PD-L1, TILs, and TLRs, are perhaps among first key elements to provide deep insight into GCT immune regulation and may certainly serve as reasonable treatment targets for future clinical trials.

## Figures and Tables

**Figure 1:**